# Engeletin Targets Mitochondrial Dysfunction to Attenuate Oxidative Stress and Experimental Colitis in Intestinal Epithelial Cells Through AMPK/SIRT1/PGC-1α Signaling

**DOI:** 10.3390/antiox14050524

**Published:** 2025-04-27

**Authors:** Jing Li, Zhijun Geng, Lixia Yin, Ju Huang, Minzhu Niu, Keni Zhang, Xue Song, Yueyue Wang, Lugen Zuo, Jianguo Hu

**Affiliations:** 1 Department of Clinical Laboratory, First Affiliated Hospital of Bengbu Medical University, Bengbu 233004, China; lijingbyfy@bbmu.edu.cn (J.L.); 20231002066@stu.bbmc.edu.cn (L.Y.); 20241002060@stu.bbmu.edu.cn (K.Z.); wangyueyue@bbmu.edu.cn (Y.W.); 2 Anhui Province Key Laboratory of Basic and Translational Research of Inflammation-Related Diseases, Bengbu 233004, China; gengzhijun1213@bbmu.edu.cn (Z.G.); 20211002062@stu.bbmu.edu.cn (J.H.); 20231001011@stu.bbmc.edu.cn (M.N.); songxue0214@bbmu.edu.cn (X.S.); zuolugen@bbmu.edu.cn (L.Z.); 3 Department of Central Laboratory, First Affiliated Hospital of Bengbu Medical University, Bengbu 233004, China; 4 Department of Gastrointestinal Surgery, First Affiliated Hospital of Bengbu Medical University, Bengbu 233004, China

**Keywords:** inflammatory bowel disease, engeletin, intestinal epithelial cells apoptosis, intestinal barrier, mitochondrial function, antioxidant

## Abstract

Inflammatory bowel disease (IBD), encompassing Crohn’s disease and ulcerative colitis, is characterized by chronic intestinal inflammation and epithelial barrier disruption. Emerging evidence highlights mitochondrial dysfunction as a pivotal contributor to IBD pathogenesis, where impaired mitochondrial homeostasis in intestinal epithelial cells (IECs) disrupts redox balance, exacerbates oxidative stress, and triggers apoptosis, further compromising barrier integrity. This study investigated the therapeutic effects of Engeletin (Eng), a dihydroflavonoid from *Smilax glabra* Roxb., in dextran sulfate sodium (DSS)-induced colitis mice and colonic organoid models. Eng administration (10, 20, 40 mg/kg) significantly alleviated colitis symptoms, including weight loss, disease activity index (DAI) scores, and colon shortening, while restoring intestinal barrier integrity through the upregulation of tight junction proteins (ZO-1, claudin-1) and goblet cell preservation. Eng suppressed NF-κB-mediated inflammation and activated the Nrf2 antioxidant pathway, as well as reduced oxidative stress markers (MDA, CAT, GSH, and SOD). It attenuated epithelial apoptosis by balancing pro- and anti-apoptotic proteins (Bax/Bcl2, c-caspase3) and ameliorated mitochondrial dysfunction via enhanced ATP production, mtDNA levels, and complex I/IV activity. Mechanistically, Eng activated the AMPK/SIRT1/PGC-1α axis, and pharmacological inhibition of PGC-1α abolished its mitochondrial protective and anti-apoptotic effects. These findings demonstrate that Eng alleviates colitis by targeting mitochondrial homeostasis and oxidative stress through AMPK/SIRT1/PGC-1α signaling, offering a multitargeted strategy for IBD therapy.

## 1. Introduction

Inflammatory bowel disease (IBD), characterized by chronic inflammation and epithelial barrier disruption, remains a therapeutic challenge due to its multifactorial pathogenesis involving dysregulated immune responses, oxidative stress, and mitochondrial dysfunction [1]. While recent studies have highlighted the role of immune cell dysregulation (e.g., M1 macrophage polarization and Helper T-cell differentiation) in IBD progression [2,3,4], intestinal epithelial cell (IEC) apoptosis and mitochondrial impairment are increasingly recognized as central drivers of barrier dysfunction and disease exacerbation [5,6,7]. Mitochondrial dysfunction in IECs drives IBD pathogenesis by disrupting redox balance, depleting ATP, and triggering apoptosis processes [8,9,10]. Despite this, pharmacological strategies that simultaneously target mitochondrial repair, oxidative stress, and inflammatory cascades in IECs remain underexplored.

The AMP-activated protein kinase (AMPK)/Sirtuin 1 (SIRT1)/Peroxisome proliferator-activated receptor gamma coactivator 1-alpha (PGC-1α) signaling axis, a master regulator of mitochondrial biogenesis and energy metabolism, has emerged as a promising therapeutic target for IBD [11]. This pathway can be activated by diverse stimuli, including pharmacological agents (e.g., metformin), physiological interventions (e.g., exercise and fasting), and natural compounds [12,13,14]. For instance, bioactive peptides combined with exercise synergistically activate AMPK/SIRT1/PGC-1α to mitigate hypertension via mitochondrial biogenesis [14], while metformin enhances antioxidant defenses in cardiac tissue independently of this pathway [13]. These findings underscore the versatility of AMPK/SIRT1/PGC-1α modulation across diseases, yet its role in natural compound-mediated IEC protection in colitis with profound implications for further research.

*Smilax glabra* Roxb. (Tu fuling), a traditional Chinese medicinal herb, has demonstrated anti-inflammatory and immunomodulatory effects in colitis models [15,16]. Engeletin (Eng), a dihydroflavonoid glycoside isolated from *Smilax glabra* Roxb., exhibits potent anti-inflammatory and antioxidant properties in diverse pathologies, including osteoarthritis and neurodegenerative diseases [17,18,19]. Critically, existing studies focus on immune cell modulation which revealed its ability to suppress Toll-like receptor 4 (TLR4)/NF-κB signaling in macrophage-mediated colitis models [20]. However, its direct effects on IEC mitochondrial homeostasis and apoptosis regulation, particularly through AMPK/SIRT1/PGC-1α signaling, remain unexplored.

In this study, we aimed to investigate whether Eng alleviates experimental colitis by restoring mitochondrial function and intestinal barrier integrity via the AMPK/SIRT1/PGC-1α pathway. Using DSS-induced colitis in mice and colonic organoid models, we systematically evaluated Eng’s effects on oxidative stress, mitochondrial function, and apoptosis. Our findings not only elucidate a novel mechanism of Eng but also highlight mitochondrial regulation as a multitargeted therapeutic paradigm for IBD.

## 2. Materials and Methods

### 2.1. Animal Experiments

**Figure 1:**
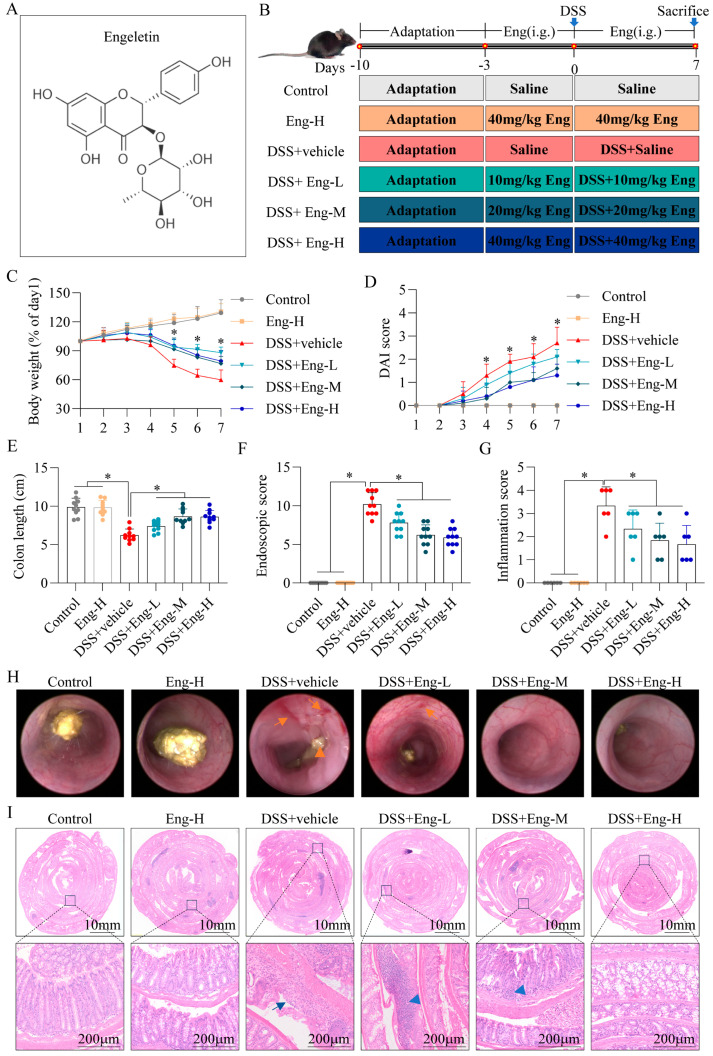
Engeletin reduces DSS-induced colonic damage in mice. (**A**) Chemical structure of Engeletin (Eng). (**B**) Experimental design timeline: Mice received daily oral administration of Eng (10, 20, or 40 mg/kg) or vehicle (corn oil) starting 3 days prior to 2.5% DSS exposure for 7 days. (**C**) Body weight changes expressed as percentage of initial weight. (**D**) Disease activity index (DAI) scores incorporating concealed blood in feces, fur folds, soft stools, and rectal prolapse. (**E**) Colon length measurements (cm) at sacrifice. (**F**) Endoscopic severity scores assessing colon thickening, vascular texture, fibrin exudation, mucosal granularity, and stool consistency. (**G**) Histopathological inflammation scores (0–4 scale) based on H&E-stained sections. (**H**) Representative endoscopic images of colonic mucosa: orange arrows indicate mucosal erosions (→) and soft stools (▲). (**I**) Swiss-roll colon sections stained with H&E: blue arrows highlight ulceration (→) and inflammatory cell infiltration (▲). Data represent mean ± SD (*n* = 10). * *p* < 0.05 vs. DSS+vehicle group (one-way ANOVA with Tukey’s post hoc test).

Wild-type (WT) mice (C57BL/6, 6–8 weeks old, male) were purchased from GemPharmatech Co., Ltd. (Nanjing, China) and acclimatized for 7 days under specific pathogen-free (SPF) environment (25 °C, 12 h light/dark cycle) with ad libitum access to standard chow and water. All experimental protocols were approved by the Animal Ethics Committee of Bengbu Medical University ([2024] No.192, Bengbu, China). Mice were randomly allocated into six groups (*n* = 10/group): (1) WT control (normal drinking water, did not receive DSS), (2) Eng-H (40 mg/kg Eng without DSS), (3) DSS+vehicle (drinking water containing the same volume of corn oil), (4) DSS+Eng-L (10 mg/kg), (5) DSS+Eng-M (20 mg/kg), and (6) DSS+Eng-H (40 mg/kg). Colitis was established by administering 2.5% (*w*/*v*) dextran sulfate sodium (DSS, molecular weight 36–50 kDa; MP Biomedicals, Santa Ana, CA, USA) in drinking water for 7 days. Engeletin (Eng, MedChemExpress, Monmouth Junction, NJ, USA; purity ≥ 99.88%, molecular formula C_21_H_22_O_10_, molecular weight 434.39) was dissolved in corn oil and administered daily via oral gavage three days before DSS exposure as outlined in Figure 1B. The determination of the intervention doses of Eng and the administration time were based on prior studies in murine models [21,22,23]. Our preliminary dose–response pilot study confirmed that 20 mg/kg achieved optimal efficacy, while higher doses (40 mg/kg) showed no additional benefits, aligning with the ‘minimal effective dose’ principle. On day 7, mice were euthanized under isoflurane anesthesia (5% induction, 2% maintenance) for tissue collection. Based on dose–response efficacy, the medium dose (Eng-M) was selected for subsequent experiments to adhere to the minimal effective dose principle.

### 2.2. Mouse Specimen Collection and Colitis Assessment

The mice were weighed, and the disease activity index (DAI) scores [24] were recorded daily prior to DSS administration. Before sacrifice, the mice were subjected to colonoscopy under anesthesia with sodium pentobarbital at a dose of 40 mg/kg via intraperitoneal injection, and the scoring criteria under endoscopy refer to the previous literatures [25,26], the eyes were removed, and serum was collected via centrifugation for analysis of intestinal fatty acid binding protein (I-FABP). The mouse’s entire colon tissue was removed and its length was measured before being divided into two parts along the longitudinal axis: one part was fixed in 10% formalin, rolled into Swiss-rolls, and embedded in paraffin for H&E staining, Alcian blue periodic acid Schiff (AB-PAS) staining and immunofluorescence detection, and the other part was removed from the intestinal mucosa tissue and divided into three parts, which were, respectively, used for quantitative reverse transcription–polymerase chain reaction (RT–qPCR), Western blotting, and enzyme-linked immunosorbent assay (ELISA) analyses. The method of making Swiss rolls of mouse intestinal were referred to reports by Min Zhang et al. [27]. According to Schultz et al., the intestinal tissues were graded on a scale of 0–4 on the basis of the degree of intestinal inflammation [28], as detailed in the Supplementary Methods (Sections S1–S4).

### 2.3. Colonic Organoid Culture and Treatment

Mouse colonic organoids were extracted and cultured according to a previously reported protocol (Supplementary Methods). In vitro experiments comprised two independent modules. In module 1, investigating Eng’s effects on DSS-induced injury, organoids were divided into six treatment groups: (1) Untreated controls (Con); (2) Eng-only (10 μM, 24 h); (3) Rotenone-only (5 μM, 24 h); (4) DSS-only (2.5%, 24 h post-starvation); (5) DSS+Eng (30-min Eng pretreatment before DSS) [29]; and (6) DSS+Eng+Rotenone (combined Eng and rotenone pretreatment before DSS). Module 2 examined AMPK/SIRT1/PGC-1α pathway involvement through two comparator groups: (1) DSS+Eng and (2) DSS+Eng+SR-18292, where organoids received additional pretreatment with the PGC-1α inhibitor SR-18292 (10 μM) prior to Eng administration and DSS exposure. Colonic organoids from all groups were collected for subsequent functional analyses of intestinal barrier integrity, mitochondrial performance, and apoptotic regulation. Colonic organoids permeability was evaluated as reported in [30]. In vitro experiments were independently repeated at least three times, and data from a representative experiment are presented.

### 2.4. Analysis of Intestinal Barrier Dysfunction

The following assays were used to assess intestinal barrier function: fluorescein isothiocyanate (FITC)-dextran (FD4; 4 kDa) permeability, serum I-FABP levels, AB-PAS staining, immunofluorescence staining, and Western blotting to evaluate both the expression and localization of tight junction proteins (details are provided in the Supplementary Methods).

### 2.5. Assessment of IECs Apoptosis

TUNEL staining, immunofluorescence staining for cleaved caspase3 (c-caspase3), and Western blot analysis of apoptosis-related proteins (c-caspase3, Bax and Bcl2) were used to assess the effects of Eng on IECs apoptosis in vivo and in vitro, as detailed in the Supplementary Methods.

### 2.6. Detection of Mitochondrial Dysfunction

Mouse IECs were isolated and cultured according to a previously reported protocol (Supplementary Methods) [31]. Additionally, the effects of Eng on mitochondrial function in IECs were assessed in DSS-induced mice by transmission electron microscopy (TEM) to evaluate the size and morphology of the mitochondria. Mitochondrial length was measured using ImageJ software (Version 1.53t; NIH, Bethesda, MD, USA) based on TEM images as reported in [32]. Briefly, TEM images were calibrated with their embedded scale bars to ensure accurate spatial resolution (µm/pixel). For each mitochondrion, a straight line was drawn along its longitudinal axis. For curved mitochondria, segmented measurements were summed to obtain total length. A minimum of 50 mitochondria per mouse were analyzed to ensure statistical robustness. Mean mitochondrial length was calculated for each mouse.

The detection methods of mitochondrial DNA (mtDNA), ATP production, and complex I and IV activity were provided in the Supplementary Methods. Moreover, the effect of Eng on the DSS-induced changes in mitochondrial function in colonic organoids was assessed via JC-1 staining, MitoTracker Red CMXRos labelling, and mtDNA and complex I and IV activity analyses (details are provided in the Supplementary Methods).

### 2.7. Western Blot Analysis of Signaling Pathways

The protein levels of p-P65, P65, Nuclear factor erythroid 2-related factor 2 (Nrf2), Heme oxygenase-1 (HO-1), NAD(P)H quinone dehydrogenase 1 (NQO1), p-AMPK, AMPK, SIRT1, and PGC-1α in mouse intestinal mucosal tissues, and organoids were analyzed by Western blotting. Detailed methods are given in the Supplementary Methods.

### 2.8. Oxidative Stress Detection

Levels of reactive oxygen species (ROS), malondialdehyde (MDA), Catalase (CAT), glutathione (GSH), and superoxide dismutase (SOD) in mouse intestinal mucosa, purified epithelial cells, and organoids were measured using assay kits (Solarbio, Beijing, China) following the manufacturer’s instructions. Detailed methods are given in the Supplementary Methods.

### 2.9. Statistical Analysis

Statistical analyses were performed using GraphPad Prism 10.0 (GraphPad Software, San Diego, CA, USA). Continuous variables with normal distribution are presented as mean ± standard deviation (SD). Differences among the six experimental groups were assessed by one-way ANOVA followed by Tukey’s post hoc test for multiple comparisons. For comparisons between two groups, a two-tailed Student’s *t*-test (parametric data) or Mann–Whitney U test (non-parametric data) was employed as appropriate. Statistical significance was defined as *p* < 0.05.

## 3. Results

### 3.1. Engeletin Reduces DSS-Induced Colonic Damage in Mice

Mice were given different doses of Eng by gavage to assess the ability of Eng to mitigate DSS-induced colitis (Figure 1A,B). Compared with those in the control group, the mice in the DSS group showed a significant decrease in body weight (Figure 1C), an increase in DAI scores (Figure 1D), and a shortened colon length (Figure 1E), whereas the colitis symptoms of the mice in the Eng intervention group were significantly improved. Additionally, colonoscopies were performed on the mice using a small animal endoscope, which revealed the presence of mucosal hyperplasia, haemorrhage, mucosal surface granularity, and the thinning of the stools in the colons of the DSS-induced mice, whereas in the Eng intervention group, the mice showed improvements in their endoscopic colon lesions and significant decreases in their scores (Figure 1F,H). The H&E staining images and inflammation scores of the colonic tissues revealed that the colon epithelial crypts of the mice in the DSS+vehicle group were severely detached, substantial inflammatory cell infiltration was observed, and their histological inflammation scores were significantly greater than those of the control group. However, Eng significantly ameliorated colonic tissue damage, especially in the high (Eng-H) and medium (Eng-M) dose groups (Figure 1G,I). Importantly, we observed that high doses of Eng had no effect on weight, DAI, colon length, or tissue damage in mice (Eng-H group). Eng ameliorates mice colitis at doses up to 40 mg/kg without observable toxicity. As the high- (Eng-H) and medium-dose (Eng-M) groups exhibited comparable efficacy, Eng-M was selected for subsequent experiments following the minimal effective dose principle.

### 3.2. Engeletin Suppresses NF-κB-Driven Inflammation and Nrf2-Regulated Oxidative Stress in DSS-Induced Colitis Mice

**Figure 2:**
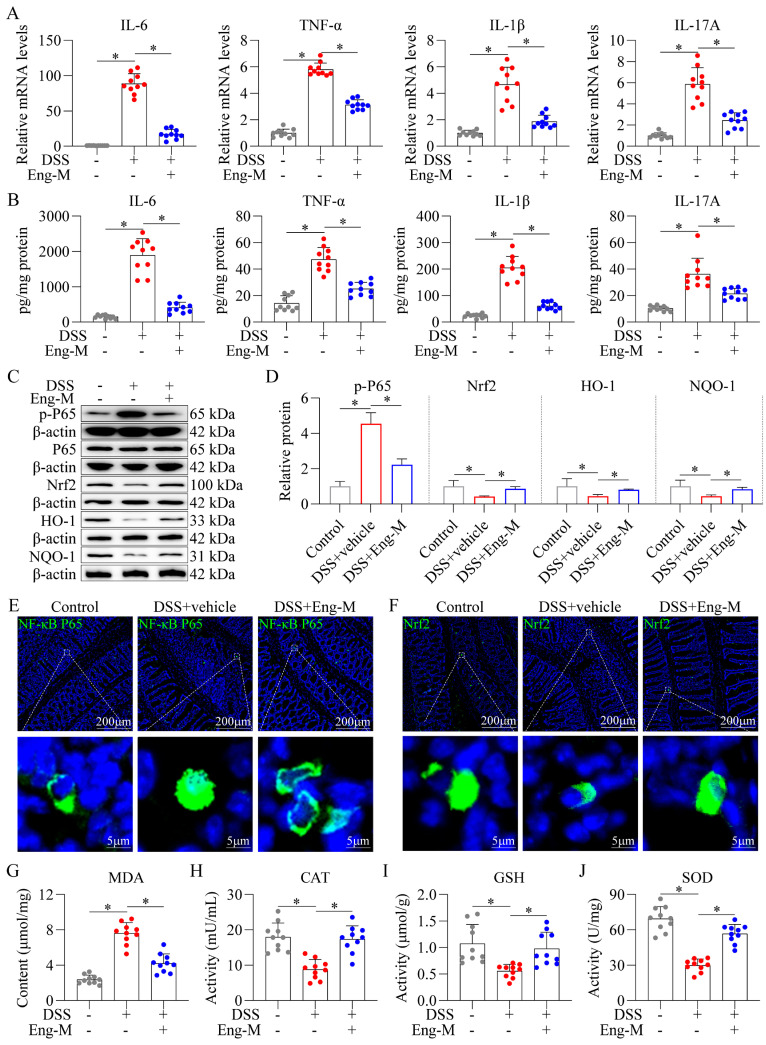
Engeletin suppresses NF-κB-driven inflammation and Nrf2-regulated oxidative stress in DSS-induced colitis mice. (**A**) The mRNA expression of pro-inflammatory cytokines (TNF-α, IL-1β, IL-6, IL-17A) in colon tissues via qRT–PCR. (**B**) Pro-inflammatory cytokines protein levels detected by ELISA. (**C**) Western blot analysis of p65, p-p65, and Nrf2 pathway proteins (Nrf2, HO-1, NQO1). (**D**) Quantification of p-p65, Nrf2, HO-1, and NQO1 band intensities normalized to β-actin. (**E**) Immunohistochemical staining of p65 nuclear translocation (Green signals). (**F**) Immunohistochemical staining of Nrf2 nuclear translocation (Green signals). (**G**) MDA content, (**H**) CAT activity, (**I**) GSH activity, and (**J**) SOD activity. Data points: Gray dots = Control group; Red dots = DSS+Vehicle group; Blue dots = DSS+Eng-M group. Data represent mean ± SD (*n* = 10). * *p* < 0.05.

Eng exerted dual regulatory effects on NF-κB-mediated inflammation and Nrf2-dependent antioxidant responses in DSS-induced colitis mice. Pro-inflammatory cytokine profiling revealed that Eng treatment (Eng-M, 20 mg/kg) significantly suppressed the DSS-induced upregulation of TNF-α, IL-1β, IL-6, and IL-17A at both the mRNA (Figure 2A) and protein level (Figure 2B). Eng abrogated NF-κB pathway activation, evidenced by the attenuated phosphorylation of p65 (Figure 2C,D), and diminished nuclear translocation of p65 in colonic epithelia (Figure 2E). Concurrently, the involvement of the Nrf2 pathway was confirmed by Western blotting results, showing that Eng enhanced protein expression of Nrf2, HO-1, and NQO1 (Figure 2C,D). Immunofluorescence further showed that Nrf2 nuclear accumulation was enhanced in the intestinal mucosa of mice in the Eng-M group (Figure 2F). Eng-M-treated mice exhibited by a reduction in lipid peroxidation (MDA content, Figure 2G), restored activities of CAT, GSH, and SOD versus DSS+vehicle group (Figure 2H–J) in intestinal tissues. These results indicate that Eng suppressed NF-κB-driven inflammation and Nrf2-regulated oxidative stress in DSS-induced colitis mice.

### 3.3. Engeletin Protects Intestinal Barrier Function In Vivo

**Figure 3:**
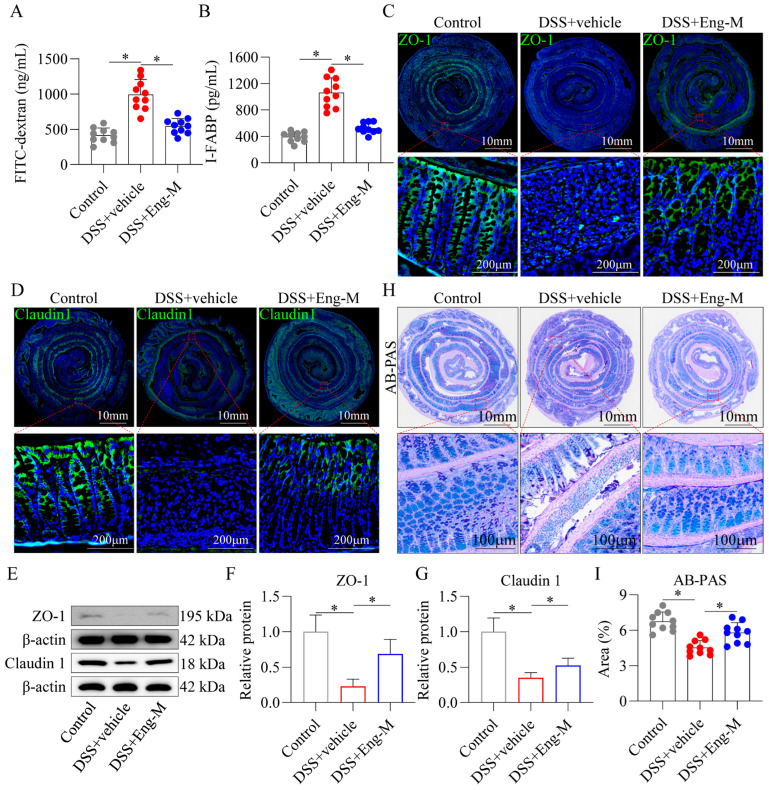
Engeletin protects intestinal barrier function in vivo. (**A**) Intestinal permeability assessed by serum FITC-dextran (FD4, 4 kDa) levels. (**B**) Serum intestinal fatty acid-binding protein (I-FABP) levels measured by ELISA. (**C**,**D**) Immunofluorescence staining of ZO-1 and Claudin-1 in colonic tissues (green: ZO-1/Claudin-1; blue: DAPI). (**E**–**G**) Western blot and quantification of ZO-1 and claudin1 expression. (**H**) Representative AB-PAS staining of mucin-positive goblet cells (light blue). (**I**) Quantification of mucin-positive area ratio. Data points: Gray dots = Control group; Red dots = DSS+Vehicle group; Blue dots = DSS+Eng-M group. Data represent mean ± SD (*n* = 10). * *p* < 0.05.

In the context of IBD, the integrity of the intestinal barrier is compromised, resulting in increased intestinal mucosa permeability, which exacerbates colitis [33]. Notably, Eng had a protective effect against DSS-induced intestinal barrier dysfunction, as indicated by the decreased serum concentrations of FITC-dextran (4 kDa) and I-FABP (Figure 3A,B). Subsequently, immunofluorescence staining revealed an aberrant distribution of the proteins ZO-1 and Claudin 1 within the colonic tissues of DSS-treated mice. This abnormal distribution was rectified following Eng intervention, as these proteins were restored to the epithelial junctions of the cell membranes (Figure 3C,D). In addition, Eng significantly upregulated ZO-1 and Claudin 1, as shown by Western blotting (Figure 3E–G). Finally, AB-PAS staining revealed a significant reduction in the number of goblet cells in the DSS group compared with the control group. Nevertheless, treatment with Eng effectively mitigated the DSS-induced reduction in goblet cell number (Figure 3H,I). These results indicate that Eng alleviated intestinal barrier injury in DSS-induced mice.

### 3.4. Engeletin Inhibits Epithelial Cell Apoptosis in DSS-Treated Mice

**Figure 4:**
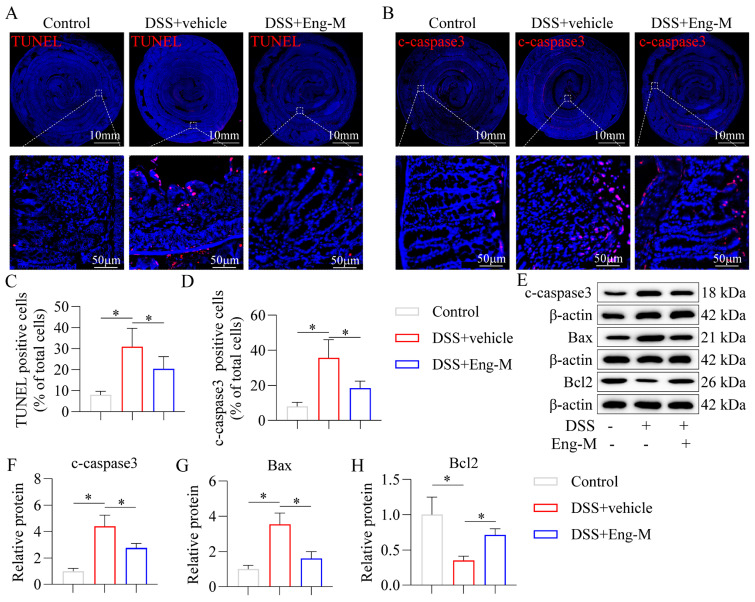
Engeletin inhibits epithelial cell apoptosis in DSS-treated mice. Fluorescence staining of (**A**) TUNEL and (**B**) cleaved caspase-3 (c-caspase-3) in colon tissues (red, apoptotic cells; blue, DAPI). (**C**,**D**) The ratios of TUNEL- and cleaved caspase 3-positive cells to the total DAPI-marked cells. (**E**–**H**) Western blotting and relative quantitative analysis of the c-caspase 3, Bax, and Bcl2 levels in the intestinal mucosa. Data represent mean ± SD (*n* = 10). * *p* < 0.05.

Persistent inflammation and high oxidative stress levels can lead to epithelial cell apoptosis, and excessive epithelial cell apoptosis is a key cause of intestinal barrier damage in IBD [34]. As shown in Figure 4A–D, Eng reduced the proportion of apoptotic IECs in DSS-induced mice, as determined by TUNEL and cleaved caspase3 staining in colonic tissues (immunofluorescence staining). In addition, Eng intervention decreased the expression of Bax and c-caspase3 and increased the level of Bcl2 compared with those in the DSS group, as confirmed by Western blotting (Figure 4E–H). Thus, the above results suggest that Eng directly inhibits IECs apoptosis in vivo.

### 3.5. Engeletin Ameliorates Mitochondrial Dysfunction and Oxidative Stress in Epithelial Cells

**Figure 5:**
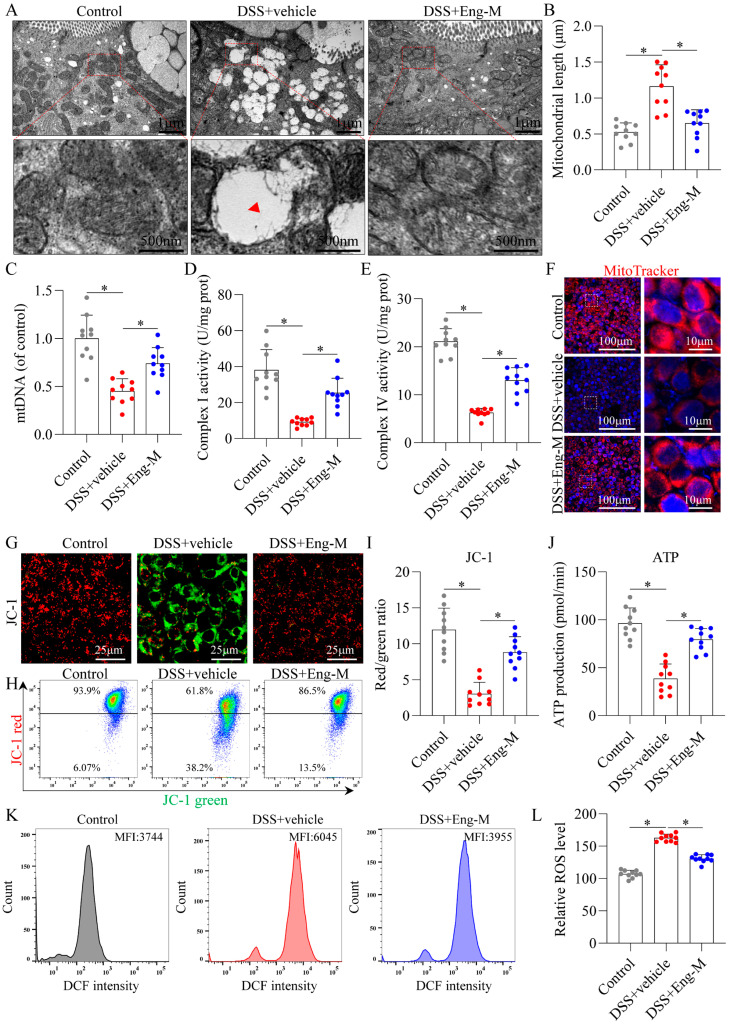
Engeletin ameliorates mitochondrial dysfunction and oxidative stress in epithelial cells. (**A**) TEM images of mitochondrial ultrastructure (red arrows, swollen mitochondria with cristae loss). (**B**) Quantitative analysis of mitochondrial length using ImageJ. (**C**) mtDNA copy number via qRT–PCR. (**D**,**E**) Respiratory chain Complex I (NADH dehydrogenase) and Complex IV (cytochrome c oxidase) activities (NADH/cytochrome c oxidation assays). (**F**) Increased mitochondrial mass via MitoTracker Green FM fluorescence. (**G**) Fluorescence staining of JC-1. (**H**) Flow cytometric profiles of JC-1 aggregates vs. monomers. (**I**) Quantification of aggregate/monomer fluorescence ratio. (**J**) ATP production in IECs. (**K**,**L**) ROS levels (DCFH-DA fluorescence intensity in IECs). Data points: Gray dots = Control group; Red dots = DSS+Vehicle group; Blue dots = DSS+Eng-M group. Data represent mean ± SD (*n* = 10). * *p* < 0.05.

Abnormal mitochondrial function leads to elevated ROS levels, which may result in increased IECs apoptosis [5,9]. TEM analysis revealed that DSS-treated group epithelia exhibited swollen mitochondria with cristae loss, whereas Eng restored near-normal mitochondrial ultrastructure comparable to WT controls (Figure 5A,B). In addition, we isolated mitochondria from mouse colon tissues and observed a decrease in the level of mtDNA and impaired complex I and complex IV activity in the DSS group, all of which were significantly reversed after Eng intervention (Figure 5C–E). Additionally, Eng improved mitochondrial function in IECs, as the mitochondrial mass (Figure 5F) and mitochondrial membrane potential increased (fewer JC-1 monomer-positive and more JC-1 aggregate-positive cells, Figure 5G–I), as shown by immunofluorescence and flow cytometry, along with the ATP production capacity (Figure 5J). In addition, Eng treatment attenuated oxidative stress, as evidenced by decreased ROS levels by DCF fluorescence intensity (Figure 5K,L). Furthermore, Eng also ameliorated mitochondrial dysfunction and increased ROS levels in DSS-induced colonic organoids (Figure S1). These results suggest that Eng ameliorated mitochondrial dysfunction in IECs in the context of colitis.

### 3.6. Engeletin Ameliorates Mitochondrial Dysfunction-Related IECs Apoptosis and Tight Junction Impairment

**Figure 6:**
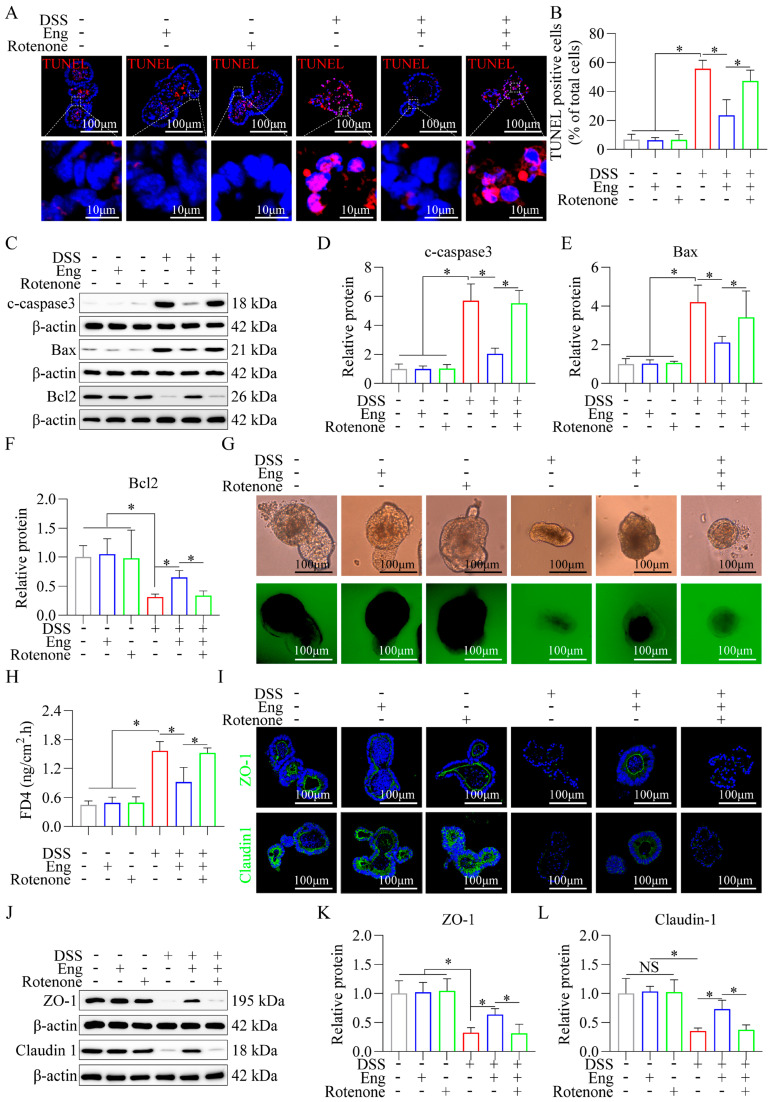
Engeletin ameliorates mitochondrial dysfunction-related IECs apoptosis and tight junction impairment. (**A**) TUNEL staining of DSS-treated colonic organoids (red, apoptotic nuclei; blue, DAPI). (**B**) Quantification of TUNEL-positive cells per field. (**C**–**F**) Representative Western blots and quantification of cleaved caspase-3 (c-caspase-3), Bax, and Bcl2. (**G**,**H**) FD4 permeability assay. (**H**) Quantitative trans-organoid flux. (**I**) Confocal imaging of ZO-1 and Claudin-1 junctional localization. (**J**–**L**) Western blot and quantification of ZO-1 and Claudin-1. The groups, from left to right, are respectively: (1) Untreated controls; (2) Eng-only (10 μM, 24 h); (3) Rotenone-only (5 μM, 24 h); (4) DSS-only (2.5%, 24 h post−starvation); (5) DSS+Eng (30−min Eng pretreatment before DSS); (6) DSS+Eng+Rotenone. Data represent mean ± SD (*n* = 3). * *p* < 0.05.

To further investigate how Eng mediates mitochondrial function in IECs for apoptosis resistance, colonic organoids were pretreated with a mitochondrial respiration inhibitor (Rotenone) prior to the administration of DSS or Eng. As shown in Figure 6A,B, the proportion of TUNEL-positive cells in the DSS-induced colonic organoids was significantly greater in the DSS+Eng+Rotenone group than in the DSS+Eng group. Additionally, Western blotting (Figure 6C–F) revealed that rotenone inhibited the effects of Eng on IECs apoptosis in DSS-induced colonic organoids, as determined upon the detection of the expression of apoptosis-related proteins (c-caspase3, Bax, and Bcl2). In addition, rotenone pretreatment inhibited the restoration of normal permeability (FD4, Figure 6G,H) and tight junction protein expression (ZO-1 and Claudin 1) in Eng-treated DSS-induced colonic organoids, as shown by immunofluorescence (Figure 6I) and Western blotting (Figure 6J–L).

These results suggest that mitochondria are involved in the mechanism by which the IECs in DSS-induced colonic organoids resist apoptosis after Eng treatment.

### 3.7. Engeletin Improves Mitochondrial Dysfunction and Reduces Apoptosis in Epithelial Cells Associated with AMPK/SIRT1 Signaling via PGC-1α-Dependent Mechanisms

**Figure 7:**
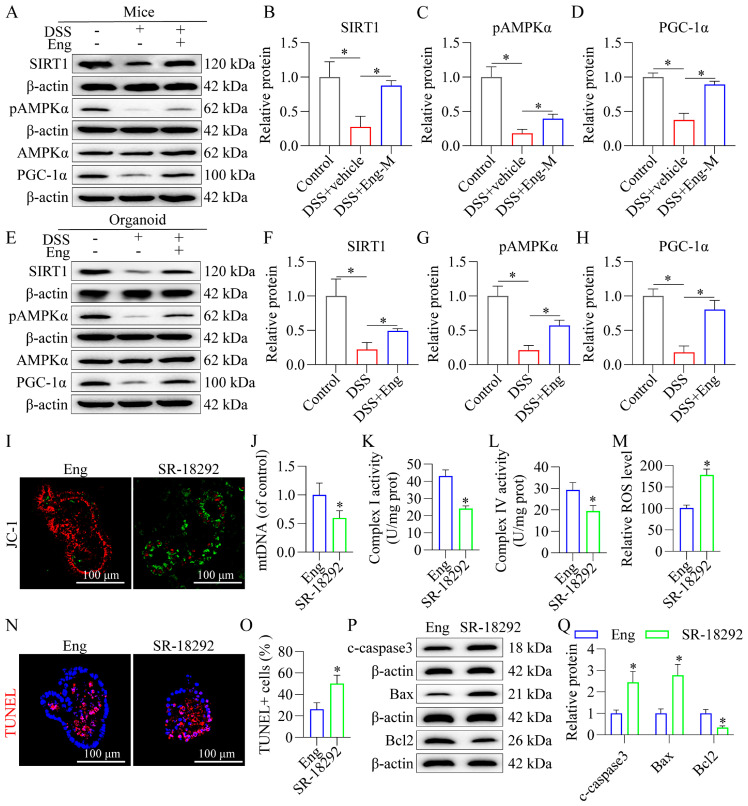
Engeletin improves mitochondrial dysfunction and reduces apoptosis in epithelial cells associated with AMPK/SIRT1 signaling via PGC-1α-dependent mechanisms. (**A**–**D**) Western blot and quantification of p-AMPK, AMPK, SIRT1, and PGC-1α in mouse intestinal mucosa. (**E**–**H**) Corresponding protein levels in colonic organoids. (**I**) Mitochondrial membrane potential in organoids as determined by fluorescence staining with the probe JC-1 (red, aggregates; green, monomers). (**J**) mtDNA levels in colonic organoids were analyzed via qRT–PCR. (**K**,**L**) Mitochondrial complex I and IV activity in colonic organoids. (**M**) ROS levels. (**N**,**O**) TUNEL staining of colonic organoids and quantification of positive cells. (**P**,**Q**) Representative Western blots and quantification of cleaved caspase 3 (c-caspase 3), Bax, and Bcl2 in the colonic organoids. *n* = 10 (in vivo) and *n* = 3 (in vitro). Data represent mean ± SD. * *p* < 0.05.

Emerging evidence implicates AMPK/SIRT1 signaling in mitochondrial homeostasis, prompting our investigation into whether Eng modulates this pathway to preserve intestinal epithelial integrity. Western blot analyses demonstrated that Eng significantly upregulated phosphorylated AMPK (p-AMPK) and SIRT1 expression in both mice colonic mucosa (Figure 7A–D) and DSS-challenged organoids (Figure 7E–H). Concomitantly, Eng enhanced the expression PGC-1α—key downstream effectors orchestrating mitochondrial biogenesis and function (Figure 7A–H). Critically, pharmacological inhibition of PGC-1α using SR-18292 abolished Eng-mediated mitochondrial protection in DSS-treated colonic organoids, as evidenced by the increase in JC-1 monomer-positive cells (green, Figure 7I), reduced mtDNA levels (Figure 7J), and decreased activities of complexes I and IV (Figure 7K,L). Notably, SR-18292 treatment abolished Eng’s antioxidative (ROS level, Figure 7M) and anti-apoptotic (Figure 7N–Q) efficacy in DSS-exposed organoids. Thus, Eng can improve apoptosis and mitochondrial function in IECs associated with the AMPK/SIRT1/PGC-1α pathway.

## 4. Discussion

This study systematically demonstrated that Eng, a flavonoid glycoside derived from *Smilax glabra* Roxb., ameliorated DSS-induced experimental colitis by preserving intestinal barrier integrity and suppressing mitochondrial dysfunction-mediated epithelial apoptosis. Mechanistically, Eng activated the AMPK/SIRT1/PGC-1α signaling axis, enhancing mitochondrial biogenesis, oxidative stress adaptation, and anti-apoptotic capacity in IECs. These effects collectively attenuated mucosal inflammation, reduced proinflammatory cytokine production (e.g., TNF-α, IL-6, IL-1β), and preserved tight junction protein expression (ZO-1 and Claudin 1), highlighting Eng as a multitargeted therapeutic candidate for IBD.

Current therapeutic strategies for IBD, including biologics targeting inflammatory cytokines (e.g., anti-TNF-α agents) and immunosuppressants, are limited by systemic side effects, variable efficacy, and failure to address underlying epithelial barrier dysfunction [35]. In contrast, natural plant-derived monomeric compounds, with their multitargeted bioactivities and favorable safety profiles, have emerged as promising candidates for IBD treatment [36]. Notably, flavonoids, such as Eng, derived from *Smilax glabra* Roxb. (Tu fuling), have demonstrated significant pharmacological value in digestive diseases by modulating inflammation, oxidative stress, and mucosal repair [18,37]. Critically, the dihydroflavonoid structure of Eng—characterized by its C2–C3 double bond saturation and glycosylation—enhances its stability and bioavailability, while the hydroxyl groups at positions 3′, 4′, and 5′ likely contribute to its potent antioxidant activity by scavenging ROS and chelating metal ions [38,39].

Prior studies highlight Eng’s therapeutic potential in colitis models, including its ability to attenuate TNBS-induced Crohn’s-like colitis via TLR4/NF-κB pathway inhibition [20]. However, our work uniquely links Eng’s efficacy to mitochondrial regulation in IECs, a mechanism distinct from its previously reported immune-centric actions. While the existing research predominantly focuses on immune cell modulation, our findings emphasize the importance of mitochondrial repair in barrier restoration, expanding Eng’s therapeutic scope [20]. Given the widespread application of Eng in metabolic and neurodegenerative disorders via antioxidant mechanisms [17,18,29], our work bridges its established bioactivities to IBD-specific mitochondrial pathology, offering a translational framework for repurposing natural compounds in epithelial-centric IBD therapies.

The DSS-induced colitis model recapitulates key pathophysiological features of human IBD, including epithelial barrier disruption, mucosal inflammation, and oxidative stress [40]. In this study, Eng demonstrated multifaceted therapeutic efficacy by alleviating colitis severity, as evidenced by reduced weight loss, normalized colon length, and improved DAI, endoscopic, and histopathological scores. Histological analysis further confirmed that Eng attenuated inflammatory cell infiltration, restored mucosal architecture, and preserved crypt integrity in DSS-challenged mice. Notably, Eng’s therapeutic benefits extended beyond immunomodulation to robust antioxidative effects. Eng significantly suppressed oxidative stress markers in colonic tissues, including reduced levels of ROS and MDA, while restoring the activities of antioxidant enzymes (CAT, SOD, and GSH). These effects were linked to the activation of the Nrf2 signaling axis, as Eng enhanced nuclear translocation of Nrf2 and upregulated its downstream targets HO-1 and NQO1—critical mediators of cellular defense against oxidative damage [41]. Additionally, Eng’s suppression of NF-κB-driven inflammation may synergize with its mitochondrial protection, as NF-κB activation is known to exacerbate mitochondrial ROS production and apoptosis in colitis models [42,43]. Concurrently, Eng inhibited the NF-κB-driven inflammatory cascade, curtailing the overexpression of proinflammatory cytokines (IL-6, TNF-α, IL-1β, and IL-17A) in intestinal mucosa, thereby disrupting the inflammatory-amplification loop characteristic of IBD [42]. Furthermore, Eng preserved intestinal barrier integrity by restoring tight junction protein (ZO-1 and Claudin 1) expression and membrane localization, reducing mucosal permeability—a hallmark of IBD pathogenesis [44]. By synergistically attenuating oxidative injury, inflammatory signaling, and barrier dysfunction, Eng exemplifies a multitargeted therapeutic agent uniquely suited for IBD management. Its ability to concurrently activate cytoprotective Nrf2 signaling and suppress NF-κB-mediated inflammation underscores its pharmacological versatility, positioning Eng as an ideal candidate for addressing the multifactorial nature of IBD.

Intriguingly, the gut microbiota—a key modulator of IBD pathogenesis—may also contribute to Eng’s therapeutic effects. Dysbiosis in IBD is closely associated with impaired mitochondrial function and epithelial apoptosis [45]. While our study did not directly evaluate microbial shifts, prior work indicates that flavonoids can restore gut microbiota diversity and reduce pathogenic bacteria (e.g., Enterobacteriaceae) [46]. Future studies should explore whether Eng’s benefits involve microbiota-mediated modulation of AMPK/SIRT1/PGC-1α signaling.

IECs play an irreplaceable role in building and maintaining the intestinal barrier by forming cellular connections, secreting mucus, regulating immune responses, absorbing nutrients, and secreting anti-microbial substances [47]. Excessive apoptosis or persistent IECs death can cause damage to and ulceration of the intestinal mucosa, which impairs intestinal barrier function and allows the passage of bacteria, toxins, and other substances across the intestinal mucosa, triggering an inflammatory response in the intestine [34,48]. Excessive IEC apoptosis is one of the main characteristics of IBD, and importantly, DSS-induced colitis model mice also show a significant increase in IEC apoptosis (as evidenced by a greater proportion of TUNEL-positive cells and abnormal expression of apoptosis-related proteins). However, our results revealed that Eng intervention can significantly inhibit the apoptosis of IECs.

Mitochondrial dysfunction, characterized by energy depletion and oxidative stress, critically compromises intestinal barrier integrity through multifaceted mechanisms: promoting epithelial apoptosis, impairing mucus secretion (notably in Paneth cells), and destabilizing tight junction networks [5]. Our findings substantiate this paradigm in DSS-induced colitis, where ultrastructural mitochondrial abnormalities (e.g., cristae disruption and matrix swelling) and diminished respiratory chain complex I/IV activities were observed in IECs. Notably, Eng treatment counteracted these defects, restoring mitochondrial mass, membrane potential, and ATP synthesis—a therapeutic effect aligning with prior reports of Eng-mediated mitochondrial rescue in osteoarthritis (chondrocytes) and Huntington’s disease models (neurons) [29,37]. While Eng’s anti-inflammatory effects have been documented in intervertebral disc degeneration (via NF-κB/MAPK suppression [18]) and Alzheimer’s disease (through Nrf2 activation [17]), our work provides the first evidence of its AMPK/SIRT1/PGC-1α axis activation in IBD. These findings position Eng as a multimodal therapeutic agent with disease-selective pathway engagement.

Specifically, Eng reversed DSS-induced mtDNA depletion in colonic mucosa, suggesting enhanced mitochondrial biogenesis—a finding further corroborated in colonic organoids where Eng’s anti-apoptotic and barrier-protective effects were abrogated upon mitochondrial respiration inhibition (Rotenone).

Mechanistically, our mechanistic studies linked these benefits to AMPK/SIRT1/PGC-1α pathway activation, a master regulator of mitochondrial homeostasis [49,50,51]. While earlier studies emphasized Eng’s anti-inflammatory properties, our work delineates its unique capacity to concurrently rectify mitochondrial bioenergetics (via AMPK-driven PGC-1α activation) and suppress apoptosis (via Bcl-2/Bax modulation), thereby disrupting the vicious cycle of mitochondrial failure and barrier breakdown. This finding contrasts with the SMYD5-mediated PGC-1α degradation pathway: whereas recent studies have shown that SMYD5 inhibits mitochondrial biosynthesis by promoting the ubiquitinated degradation of PGC-1α through methylation modifications [52], the present study found that Eng enhances mitochondrial biogenesis through activation of the AMPK/SIRT1/PGC-1α axis, suggesting that it may be able to maintain mitochondrial biosynthesis through the antagonism of the methylation of SMYD5 to maintain PGC-1α stability. This mechanism was further validated in a colonic organoid model—when mitochondrial respiration was inhibited by rotenone, the anti-apoptotic and barrier-protective effects of Eng were significantly reduced. This dual targeting of epithelial survival and metabolic resilience distinguishes Eng from conventional IBD therapies, positioning it as a multitiered therapeutic agent capable of addressing both upstream mitochondrial pathology and downstream barrier dysfunction in IBD.

While this study demonstrates Eng’s potential to mitigate experimental colitis via AMPK/SIRT1/PGC-1α-mediated mitochondrial restoration, certain limitations highlight areas beyond the current scope. First, our findings are derived from a chemically induced acute colitis model (DSS), which predominantly reflects epithelial injury-driven inflammation but does not fully recapitulate the immune dysregulation or chronicity observed in human Crohn’s disease (CD) or ulcerative colitis (UC). Although DSS models are widely utilized, complementary validation in chronic models (e.g., IL-10^−/−^ mice) or immune-centric systems is needed to generalize Eng’s therapeutic applicability. Second, while we confirmed the PGC-1α signaling and mitochondrial involvement through functional assays (e.g., rotenone inhibition); the upstream mechanisms linking Eng to AMPK/SIRT1 activation remain unresolved. Whether Eng directly engages these targets or acts via intermediate metabolites requires deeper pharmacological interrogation. Third, the absence of human colonoid data—particularly from IBD patients—limits the translational relevance of our findings. Human colonoids would provide critical insights into Eng’s effects on patient-specific epithelial pathophysiology. Fourth, despite its multitarget bioactivity as a flavone glycoside, our study focused narrowly on mitochondrial protection and apoptosis regulation, leaving unexplored its potential immunomodulatory or neuroprotective roles in the multicellular intestinal microenvironment. Lastly, while epithelial-centric mechanisms were rigorously investigated, IBD pathogenesis involves intricate epithelial–immune–stromal crosstalk, necessitating future studies using organoid-immune co-cultures or cell-type-specific models to dissect Eng’s broader cellular interactions. While this study demonstrates Eng’s efficacy in preventing colitis development, its applicability to human IBD requires careful consideration. Given the unpredictable onset of human IBD, prophylactic administration (as implemented here) may primarily benefit high-risk populations (e.g., genetic predisposition or remission maintenance). However, the more clinically urgent need lies in therapeutic interventions for active disease. Future studies comparing prophylactic (pre-DSS) versus therapeutic (post-DSS-onset) regimens will clarify their translational potential, particularly regarding dose optimization and treatment window. These gaps represent natural constraints of hypothesis-driven research rather than fundamental flaws, underscoring opportunities for targeted follow-up investigations.

## 5. Conclusions

In conclusion, Engeletin emerges as a novel multitarget therapeutic agent for IBD by restoring mitochondrial homeostasis and suppressing epithelial apoptosis via AMPK/SIRT1/PGC-1α signaling. It alleviates colitis by balancing NF-κB-mediated inflammation and Nrf2-driven antioxidant defenses while reinforcing intestinal barrier integrity through tight junction preservation and goblet cell restoration. Mitochondrial functional recovery—evidenced by improved bioenergetics and reduced oxidative stress—underpins its anti-apoptotic efficacy, with AMPK/SIRT1/PGC-1α activation identified as the central mechanistic driver. These findings not only establish mitochondrial functional recovery as a critical therapeutic strategy in IBD but also highlight Eng’s translational promise as a natural compound bridging epithelial protection and metabolic regulation.

## Data Availability

The data used to support the findings of this study are available from the corresponding author upon request.

## Supplementary Materials

The following supporting information can be downloaded at: https://www.mdpi.com/article/10.3390/antiox14050524/s1. Supplementary Methods: S1. Colonoscopy scores, S2. Disease activity index (DAI) score, S3. Haematoxylin and eosin (H&E) staining, S4. Alcian blue periodic acid Schiff (AB–PAS) staining, S5. Colonic organoid extraction and culture, S6. Isolation of mouse primary colonic intestinal epithelial cells, S7. qRT–PCR analysis, S8. Enzyme-linked immunosorbent assay (ELISA), S9. Western blotting, S10. Intestinal barrier permeability analysis, S11. Immunofluorescence analysis, S12. TUNEL assay, S13. Transmission electron microscopy (TEM) analysis, S14. Quantitative analysis of the mitochondrial DNA (mtDNA) copy numbers, S15. Detection of the activities of mitochondrial complexes I and IV, S16. JC-1 assay, S17. MitoTracker Red CMXRos labelling, S18. ATP Measurement, S19. Reactive oxygen species (ROS) Detection, S20. Biochemical Quantification of Antioxidant/Oxidative Stress Parameters. Supplementary tables. Table S1. Endoscopy scoring criteria, Table S2. Sequences of primers used in this study. Figure S1. Engeletin ameliorated mitochondrial dysfunction in DSS-induced colonic organoid. (A) The mitochondrial membrane potential in colonic organoids was assessed by fluorescence staining with the probe JC-1. (B) mtDNA levels in mitochondria isolated from colonic organoids were analyzed via qRT–PCR. (C,D) Mitochondrial complex I and IV activity in colonic organoids. (E,F) ROS levels (DCFH-DA fluorescence intensity in colonic organoid). Data represent mean ± SD (*n* = 3). * *p* < 0.05.

## Abbreviations

IBD, Inflammatory bowel disease; CD, Crohn’s disease; UC, Ulcerative colitis; Eng, Engeletin; IEC, Intestinal epithelial cell; ROS, Reactive oxygen species; DSS, Dextran sodium sulfate; WT, Wild-type; H&E, Haematoxylin and eosin; SPF, Specific pathogen-free; DAI, Disease activity index; ELISA, Enzyme-linked immunosorbent assay; TEM, Transmission electron microscopy; mtDNA, Mitochondrial DNA; ATP, Adenosine triphosphate; MDA, Malondialdehyde; CAT, Catalase; GSH, Glutathione S-transferase; SOD, Superoxide dismutase; NF-κB, Nuclear factor kappa-light-chain-enhancer of activated B cells; Nrf2, Nuclear factor erythroid 2-related factor 2; HO-1, Heme oxygenase-1; NQO1, NAD(P)H quinone dehydrogenase 1; AMPK, AMP-activated protein kinase; SIRT1, Sirtuin 1; PGC-1α, Peroxisome proliferator-activated receptor gamma coactivator 1-alpha; SR-18292, PGC-1α inhibitor; TNF-α, Tumor necrosis factor-alpha; IL-1β, Interleukin-1 beta; IL-6, Interleukin-6; IL-17A, Interleukin-17A; ZO-1, Zonula occludens-1; Bax, Bcl-2-associated X protein; Bcl2, B-cell lymphoma 2; FD4, Fluorescein isothiocyanate-dextran 4 kDa; I-FABP, Intestinal fatty acid binding protein; AB-PAS, Alcian blue periodic acid Schiff; TLR4, Toll-like receptor 4; TNBS, Trinitrobenzene sulfonic acid; SMYD5, SET and MYND domain-containing protein 5.
