# Supplementary material for: Engeletin Targets Mitochondrial Dysfunction to Attenuate Oxidative Stress and Experimental Colitis in Intestinal Epithelial Cells Through AMPK/SIRT1/PGC-1α Signaling
Source: Antioxidants (Basel). 2025 Apr 27;14(5):524. doi: 10.3390/antiox14050524 (PMC12108241; doi:10.3390/antiox14050524)
Supplement: Supplementary file 1 [file antioxidants-14-00524-s001.zip › antioxidants-3578714-supplementary.pdf]

Supplementary Methods

S1. Colonoscopy scores

The mice were anaesthetized and subsequently laid flat atop a heating pad. Employing a sterile swab, the anus widened slightly. The subject was held securely with the left hand, and the colonoscope was deftly introduced using the right hand. The inflammatory conditions of the intestinal lining were captured and documented via video recording. On the basis of the findings from the colonoscopy, the following scoring system with five criteria was applied to evaluate the state of the colon: mucosal granularity, vascular texture, presence of fibrin, stool characteristics, and colon thickness; the maximum cumulative score was 15 points. The specific scoring parameters are detailed in Table S1.

S2. Disease activity index (DAI) score

The numerical system described by Spencer *et al.* was used to determine the inflammatory bowel DAI in mice with DSS-induced colitis. The DAI was determined by assessing concealed blood in feces, fur folds, soft stools, and rectal prolapse (<1 mm), and the presence of each feature was given a score of 1 point. An additional point was added when severe rectal prolapse exceeding 1 mm or diarrhea was present. The DAI was determined on a 6-point scale (0–5).

S3. Haematoxylin and eosin (H&E) staining

Colon tissue was fixed in 10% formalin and incised longitudinally, excess fluid was gently removed with absorbent paper, and the unfolded intestinal tissue was tightly rolled into a "Swiss roll" and embedded in paraffin. The paraffin–embedded tissues were sectioned at a thickness of 4 μm and stained with H&E (Solarbio, Beijing, China). Automated staining (Leica ST5010, Germany) was performed with standardized protocols: hematoxylin immersion for 10 min and eosin counterstaining for 3 min. Pathological images were captured using SlideView VS200 (OLYMPUS, Japan). According to Schultz

et al., the intestinal tissues were graded on a scale of 0-4 on the basis of the degree of intestinal inflammation. H&E staining was performed on full colon sections, and the entire tissue area was analyzed using ImageJ (NIH). All the samples were scored by 2 independent pathologists who were blinded to the treatments.

S4. Alcian blue periodic acid Schiff (AB–PAS) staining

Commencing with dewaxing through xylene-ethanol gradients to ddH_2O, sections undergo sequential processing: (1) Immersion in Alcian Blue working solution (Solarbio) for acidic mucopolysaccharide detection (10 min), followed by triple ddH_2O rinses (1 min each). (2) Oxidation in 0.5% periodic acid solution (5 min) with dual ddH_2O washes. (3) Schiff reagent incubation under light-protected conditions (10 min), terminated by running water flush. (4) Nuclear counterstaining with Harris hematoxylin (1 min) and bluing in Scott's solution (3 min). (5) Brief differentiation in 1% acid-alcohol (2 sec) with immediate aqueous neutralization. Following dehydration with an ethanol series, clearing with xylene and sealing with resin, pathological images were captured using a SlideView VS200 (OLYMPUS). AB–PAS staining was performed on full colon sections, and the entire tissue area was analyzed using ImageJ (NIH).

S5. Colonic organoid extraction and culture

As previously described, mice were sterilized with medical alcohol for 30 min after sacrifice. The colon was removed, isolated, cut longitudinally to remove the faeces, connective tissue and adipose tissue, cut into approximately 5 mm pieces, and washed with cold phosphate–buffered saline (PBS). After the PBS was discarded, 10 mL of Mild Cell Dissociation Reagent (STEMCELL Technologies, Vancouver, Canada) was added, and the mixture was incubated on ice for 30 min. The supernatant was discarded after centrifugation (2500 rpm, 5 min), and precooled 1% bovine serum albumin (BSA) (5 mL, Sigma) was added to resuspend the isolated precipitate, which was then filtered through a 70 μm filter for crypt

enrichment. Using pre-chilled tips, 50 μL Matrigel (Corning, NY, USA)-crypt mixtures (containing 100 crypts) were plated as central domes in 24-well plates. After polymerization (37°C, 1 h), 600 μL murine organoid medium (STEMCELL Technologies) was added, with medium renewal every 72 h.

S6. Isolation of mouse primary colonic intestinal epithelial cells

The colons of the mice were removed, opened longitudinally, and rinsed three times thoroughly with DPBS. Each sample was then transferred to D-Hank's buffer containing 1 mM dithiothreitol (DTT) and incubated for 10 min at 37°C with shaking. The supernatant was discarded and replaced with fresh D-Hank's buffer containing 2 mM DTT for 20 min of incubation at 37°C with shaking. The supernatant was collected, and the intestine was rinsed three times with DPBS. All the collected supernatants were passed through a 100 μm cell strainer to obtain single-cell suspensions, which were collected as the intestinal epithelial cell fraction. The intestinal epithelial cell fraction was isolated to explore Engeletin's role in ameliorating mitochondrial dysfunction and oxidative stress.

S7. qRT–PCR analysis

Total RNA was isolated from the mouse colon tissue or colonic organoids using a TRIzol total RNA isolation system. One microgram of total RNA was used for cDNA synthesis with a cDNA synthesis system kit (TaKaRa, Japan). The TB Green® Premix Ex Taq™ II detection system (TaKaRa) was used for real-time PCR according to the manufacturer's instructions. Relative gene expression normalized to GAPDH was calculated via $2^{-\Delta\Delta CT}$ method, and the mouse-specific primers used are shown in Table S2.

S8. Enzyme-linked immunosorbent assay (ELISA)

Mouse colon tissue was homogenized in 1 mL of saline containing protease inhibitors (Roche Applied Science), and the mouse intestinal organoid medium supernatants were collected. The samples were centrifuged at 1000 × g for 30 min, and the levels of interleukin (IL)-6, tumor necrosis factor-α (TNF-α),

IL-1β and IL-17A were measured via ELISA according to the manufacturer's instructions (BOSTER, Wuhan, China). Mouse peripheral blood was obtained from the eyeball, and after centrifugation (400 g, 10 min), the supernatant was collected to detect intestinal fatty acid binding protein (I-FABP) via ELISA according to the manufacturer's instructions (BOSTER, Wuhan, China).

S9. Western blotting

Mouse colon tissue or colonic organoids were fully lysed, the supernatant was collected by centrifugation and denatured by boiling, and the proteins were separated and transferred to a PVDF membrane (Millipore, Massachusetts, USA). The membrane was incubated with anti-p-P65 (Abcam, Cambridge, UK; 1:1000), anti-P65 (Abcam, 1:1000), anti-HO-1 (Abcam, 1:1000), anti-NQO1 (Abcam, 1:1000) anti-ZO1 (Stanta Cruz Biotechnology, Texas, USA; 1:400), anti-claudin1 (Abcam, 1:1000), anti-cleaved caspase 3 (Abcam, 1:1000), anti-Bax (Abcam, 1:1000), anti-Bcl2 (Abcam, 1:1000), anti-p-AMPKα (Abcam, 1:1000), anti-AMPKα (Abcam, 1:1000), anti-SIRT1 (Abcam, 1:1000), anti-PGC-1α (Abcam, 1:1000), anti-β-actin (Abcam, 1:1000), and HRP-conjugated goat anti-mouse/rabbit IgG. Finally, an imager (ChemiDoc MP, Bio-Rad, USA) was used for visualization. Relative quantitative analysis of band intensities was performed using ImageJ software, with β-actin serving as an internal reference for normalization.

S10. Intestinal barrier permeability analysis

The mice were fasted for 4 h and then administered fluorescein isothiocyanate (FITC)-dextran (FD4; 600 mg/kg, Sigma–Aldrich). Blood was obtained after 4 h by cardiac puncture, the serum was separated via centrifuged at 3,000×g for 10 min, and the serum FITC levels were assessed via fluorometry.

As described previously, colonic organoids permeability was evaluated by adding 0.1 mg/mL FD4 to the growth media and measuring the flux of FD4 into the colonic organoids with a well-developed columnar

epithelium 24 h later. The fluorescence intensity at the midpoint of the organoid lumen was determined by examining a series of optical slices through each organoid using an OLYMPUS FV3000 confocal microscope (Japan). The fluorescence intensity was then converted to a concentration using the standard curves created from log serial dilutions of FD4 in growth media. The surface area and volume of the colonic organoids were calculated from the measured radius to calculate the FD4 flux ($ng/cm^2.h$). The radius of individual colonoids was measured from the maximum cross-sectional area of Z-stack images using ImageJ software (OLYMPUS).

S11. Immunofluorescence analysis

Mouse colon tissue and colonic organoid sections were deparaffinized, antigen-exposed, blocked, incubated with anti-P65 (Abcam, 1:400), anti-Nrf2 (Abcam, 1:400), anti-ZO-1 (Stanta Cruz Biotechnology, 1:200), anti-claudin1 (Abcam, 1:400), and anti-cleaved caspase-3 (Abcam, 1:200) antibodies and then incubated with FITC-conjugated goat anti-rabbit IgG (H+L) (1:500) and/or Alexa Fluor® 555-conjugated goat anti-mouse IgG (H+L) (1:500). Finally, the nuclei were stained with DAPI. The immunofluorescence images were obtained using a Leica THUNDER LED8 (Germany) with LAS X 3.7 software under controlled excitation: FITC (488 nm), Alexa 555 (561 nm), DAPI (405 nm).

S12. TUNEL assay

TUNEL assays were performed using the In Situ Cell Death Assay Kit (Roche Applied Science) according to the manufacturer's instructions. Briefly, after dewaxing and hydration, 4-μm-thick colonic organoid sections were incubated with 0.3% Triton X-100 for 10 min at room temperature. The sections were incubated with TUNEL reaction mixture at 37°C for 1 h, and the nuclei were stained with DAPI. TUNEL fluorescence images were captured using a Leica THUNDER LED8 (Ex=593 nm, Em=614 nm).

S13. Transmission electron microscopy (TEM) analysis

As previously described, mouse colon tissues were fixed with glutaraldehyde, cut into small pieces and sectioned. The ultrastructure of the tissues was observed using a 75 kV transmission electron microscope (Tokyo, Japan), and the length of the mitochondria was measured using ImageJ software.

S14. Quantitative analysis of the mitochondrial DNA (mtDNA) copy numbers

mtDNA was extracted from mouse intestinal mucosal tissues and colonic organoids via the Mitochondrial DNA Mitochondrial Kit (BioVision, CA, USA) according to the manufacturer's instructions. Then, relative mtDNA quantification was performed via qPCR, using β-globin as an internal reference and the specific primers shown in Table S2.

S15. Detection of the activities of mitochondrial complexes I and IV

Mitochondria were extracted from mouse intestinal mucosal tissues and colonic organoids using the Mitochondrial Isolation Kit (Beyotime, Beijing, China), and the activities of complex I and complex IV in mitochondria were subsequently assessed using the Complex I Activity Assay Kit and Complex IV Activity Assay Kit (Beyotime).

S16. JC-1 assay

Isolated mouse intestinal epithelial cells and colonic organoids were made into cell slides, incubated with 500 μL of JC-1 staining working solution (Servicebio, Wuhan, China) at 37°C for 20 min, and washed with JC-1 buffer. Then, the JC-1 monomers (Ex=490 nm, Em=525 nm) and aggregates (Ex=525 nm, Em=590 nm) were examined via flow cytometry (FACSCanto, BD) or laser confocal microscopy (FV3000, OLYMPUS).

S17. MitoTracker Red CMXRos labelling

Colonic organoids were washed with prewarmed buffer and incubated with prewarmed 0.2 μM

MitoTracker Red CMXRos working solution (Servicebio) for 30 min at 37°C. After washing again with buffer, the nuclei were stained with DAPI, and the samples were placed under a confocal microscope (FV3000, OLYMPUS) for image collection (Ex=579 nm， Em=599 nm).

S18. ATP Measurement

Intracellular ATP levels in purified murine intestinal epithelial cells were determined using the ATP Assay Kit (Beyotime Biotechnology, China). Cells (1×10^5/sample) were pelleted by centrifugation, resuspended in 200 μL ice-cold lysis buffer, and vortexed thoroughly to ensure complete lysis. Lysates were centrifuged at 12,000×g for 5 min at 4°C, and supernatants were collected. A standard curve (0.01–10 μM ATP) was freshly prepared by serial dilution of ATP stock solution with lysis buffer. The ATP detection working solution was prepared by diluting the luciferase reagent 1:9 with assay buffer and stored on ice. For measurement, 100 μL of working solution was added to each well of an opaque 96-well plate, incubated at room temperature for 5 min to eliminate background ATP, followed by addition of 20 μL sample or standard. Luminescence was immediately recorded using a SpectraMax M5 microplate reader (Molecular Devices) after 2-sec mixing. ATP concentrations were normalized to total protein quantified via BCA assay. All procedures were performed on ice or at 4°C unless specified.

S19. Reactive oxygen species (ROS) Detection

Intestinal epithelial cells were plated in 24-well plates (2×10^5 cells/well) and cultured overnight at 37 °C under 5% CO_2. Cells were exposed to 2.5% dextran sulfate sodium (DSS) and pretreatment with or without 10 μM Eng for 24 h. After treatment, cells were washed twice with ice-cold PBS and incubated with 2 μM 2′,7′-dichlorodihydrofluorescein diacetate (DCFH-DA) for 15 min at 37 °C. Fluorescent images were captured using a confocal (Olympus, Japan), while intracellular ROS levels were quantitatively assessed via flow cytometry (BD Biosciences). All procedures adhered to standardized

protocols to minimize experimental variability.

S20. Biochemical Quantification of Antioxidant/Oxidative Stress Parameters

Antioxidant enzyme activities (superoxide dismutase, SOD; catalase, CAT) and oxidative stress markers (glutathione, GSH; malondialdehyde, MDA) were analyzed using commercially available kits (BioVision, Inc., California, USA). All assays were performed in strict accordance with the manufacturer's protocols to ensure standardized quantification of biochemical parameters.

Supplementary tables

Table S1. Endoscopy scoring criteria

Score	Colon thickening	Vascular texture	Fibrin exudation	Mucosal granularity	Stool consistency
0	Transparent	Normal	None	None	Hard
1	Moderate	Moderate	Little	Moderate	Soft
2	Marked	Marked	Marked	Marked	Unshaped
3	Nontransparent	Absent	Extreme	Extreme	Spread

Table S2. Sequences of primers used in this study

Primer name	Gene ID	Forwards (5'-3')	Reverse (5'-3')
IL-6	16193	TCTATACCACTTCACAAGTCGGA	GAATTGCCATTGCACAACTCTTT
TNF-α	21926	CAGGCGGTGCCTATGTCTC	CGATCACCCCGAAGTTCAGTAG
IL-1β	16176	GAAATGCCACCTTTTGACAGTG	TGGATGCTCTCATCAGGACAG
IL-17A	16171	GGCCCTCAGACTACCTCAAC	TCTCGACCCTGAAAGTGAAGG
GAPDH	14433	TGGCCTTCCGTGTTCCTAC	GAGTTGCTGTTGAAGTCGCA
mtDNA	226153	ACACCAAGGTTAATGTAGC	TTGAATCCATCTAAGCATT
β-globin	15132	CAGTACTTTAAGTTGGAAACG	ATCAACATAATTGCAGAGC

Figure S1. Engeletin ameliorated mitochondrial dysfunction in DSS-induced colonic organoid.

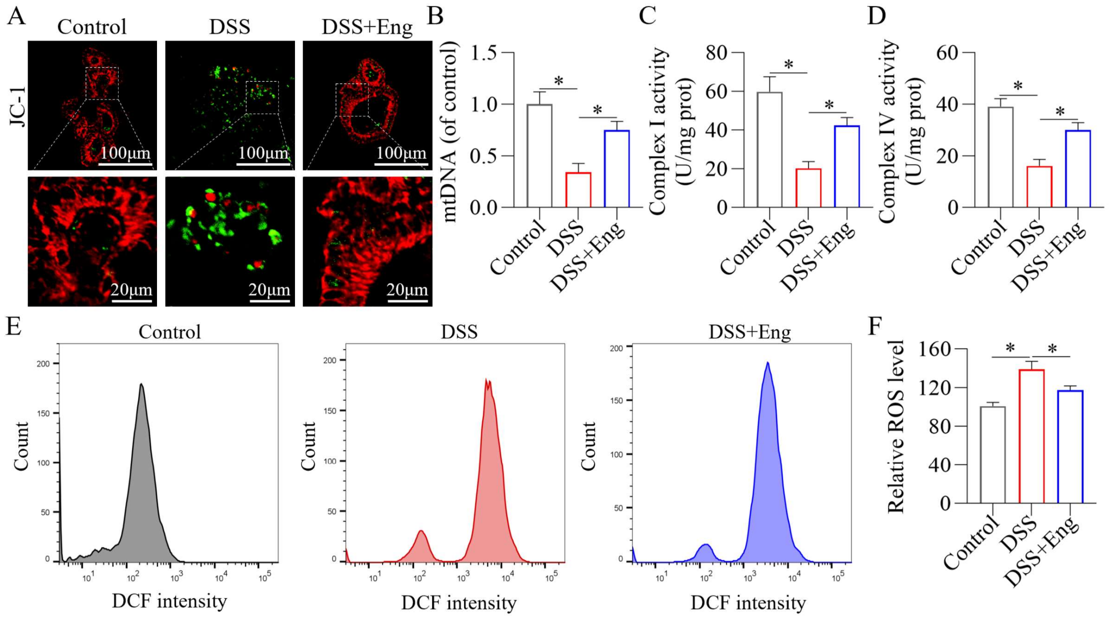

(A) The mitochondrial membrane potential in colonic organoids was assessed by fluorescence staining with the probe JC-1. (B) mtDNA levels in mitochondria isolated from colonic organoids were analyzed via qRT–PCR. (C-D) Mitochondrial complex I and IV activity in colonic organoids. (E-F) ROS levels (DCFH-DA fluorescence intensity in colonic organoid). Data represent mean ± SD (n=3). *P<0.05.
